# Ethnicity classification systems for public health surveys: experiences from HIV behavioural surveillance among men who have sex with men

**DOI:** 10.1186/s12889-020-09517-4

**Published:** 2020-09-21

**Authors:** Nathan J. Lachowsky, Peter J.W. Saxton, Nigel P. Dickson, Anthony J. Hughes, Rhys G. Jones, Terryann C. Clark, Elsie Ho, Alastair J.S. Summerlee, Cate E. Dewey

**Affiliations:** 1 School of Public Health and Social Policy, Faculty of Human and Social Development, University of Victoria, Victoria, BC V8Q 2Y2 Canada; 2 School of Population Health, Faculty of Medical and Health Sciences, University of Auckland, Auckland, 1023 New Zealand; 3 Department of Preventive and Social Medicine, Dunedin School of Medicine, University of Otago, Dunedin, 9054 New Zealand; 4 New Zealand AIDS Foundation, Auckland, 1011 New Zealand; 5 Te Kupenga Hauora Māori (TKHM), Faculty of Medical and Health Sciences, University of Auckland, Auckland, 1072 New Zealand; 6 School of Nursing, Faculty of Medical and Health Sciences, University of Auckland, Auckland, 1142 New Zealand; 7 Social and Community Health, Faculty of Medical and Health Sciences, University of Auckland, Auckland, 1072 New Zealand; 8 Department of Biomedical Sciences, University of Guelph, Guelph, ON N1G 2W1 Canada; 9 Department of Population Medicine, University of Guelph, Guelph, ON N1G 2W1 Canada

**Keywords:** Ethnicity classification, Public health, Surveillance, Sexual health, Health equity, Race, Racism, Surveys, Men who have sex with men (MSM), New Zealand

## Abstract

**Background:**

Race and ethnicity classification systems have considerable implications for public health, including the potential to reveal or mask inequities. Given increasing “super-diversity” and multiple racial/ethnic identities in many global settings, especially among younger generations, different ethnicity classification systems can underrepresent population heterogeneity and can misallocate and render invisible Indigenous people and ethnic minorities. We investigated three ethnicity classification methods and their relationship to sample size, socio-demographics and sexual health indicators.

**Methods:**

We examined data from New Zealand’s HIV behavioural surveillance programme for men who have sex with men (MSM) in 2006, 2008, 2011, and 2014. Participation was voluntary, anonymous and self-completed; recruitment was via community venues and online. Ethnicity allowed for multiple responses; we investigated three methods of dealing with these: *Prioritisation*, *Single/Combination*, and *Total Response*. Major ethnic groups included Asian, European, indigenous Māori, and Pacific. For each classification method, statistically significant associations with ethnicity for demographic and eight sexual health indicators were assessed using multivariable logistic regression.

**Results:**

Overall, 10,525 MSM provided ethnicity data. Classification methods produced different sample sizes, and there were ethnic disparities for every sexual health indicator. In multivariable analysis, when compared with European MSM, ethnic differences were inconsistent across classification systems for two of the eight sexual health outcomes: Māori MSM were less likely to report regular partner condomless anal intercourse using *Prioritisation* or *Total Response* but not *Single/Combination*, and Pacific MSM were more likely to report an STI diagnosis when using *Total Response* but not *Prioritisation* or *Single/Combination*.

**Conclusions:**

Different classification approaches alter sample sizes and identification of health inequities. Future research should strive for equal explanatory power of Indigenous and ethnic minority groups and examine additional measures such as socially-assigned ethnicity and experiences of discrimination and racism. These findings have broad implications for surveillance and research that is used to inform public health responses.

## Background

Race and ethnicity identity classification systems have considerable implications for public health. Surveillance data are frequently used to identify disparities between racial/ethnic groups and to monitor trends [1, 2]. Ethnic and racial identities are often used as a proxy for the impact of social processes of racialization such as experiences of racism. The systems used to categorise respondents into race/ethnicity variables have potential to reveal or mask important public health inequities and therefore require attention. Not only do racial/ethnicity classification systems influence research processes such as data collection, analysis, and reporting, but the outcomes of these systems can also activate public health responses, allocate resources, and stimulate further inquiry. As such, poor applications of these systems can undermine social justice-informed public health research and practice, but if used meaningfully, they have the power to redress ethnoracial discrimination and ameliorate health and social inequities by race and ethnicity.

In the human immunodeficiency virus (HIV) field, for example, differences in health outcomes and behaviours between Black men who have sex with men (MSM) and non-Black MSM were reported in the United States, Canada, and the United Kingdom [3]. This review identified that elimination of these disparities would not be achieved without addressing structural barriers such as unemployment, low income, incarceration, and low education [3]. Consequently, race and ethnicity information is an important feature of country-level responses to HIV and sexually transmitted infections (STI) and is fundamental to monitoring progress on social justice objectives [1]. However, few studies have empirically compared alternative racial/ethnicity classification systems used in public health research or surveillance [4, 5] to understand better whether they influence findings, how, and for whom.

Given increasing “superdiversity”, which describes enhanced levels of ethnoracial diversity as a result of contemporary immigration, [6] and multiple racial/ethnic identities in many global settings, especially among younger generations, different ethnicity classification systems can underrepresent population heterogeneity and can misallocate and render invisible Indigenous people and ethnic minorities. Aotearoa New Zealand offers a unique setting to examine these issues. Ethnicity variables are routinely included in public health research as mandated by crown responsibilities under the Treaty of Waitangi, New Zealand’s founding document [7, 8]. In New Zealand, ethnicity is a measure of cultural affiliation (self-identity), and is not a measure of race, ancestry, nationality, or citizenship. Ethnicity data in New Zealand are commonly collected using the national census question that allows for multiple responses. Three optional classification systems or methods have been developed to deal with this: prioritisation, single/combination, and total response [9]. Like Canada and Australia, New Zealand’s HIV epidemic is concentrated among MSM, but, unlike these countries, Indigenous MSM are not overrepresented in HIV diagnosis statistics [10]. New Zealand also has an established programme of HIV behavioural surveillance among MSM with large and diverse samples, [2] enabling us to explore the effect of different classification systems on ethnoracial disparities in HIV risk and health seeking behaviours.

The overall objective of the current paper is to provide a quantitative evidentiary foundation for future methodological consideration and use of ethnicity data. Our aim was to investigate three different ethnicity classification methods (prioritisation, single/combination, and total response) and their impact on the sample size, resulting demographics, and eight behavioural and health screening indicators among MSM recruited into HIV behavioural surveillance in New Zealand. Although focused on HIV, we believe this study has implications for all medical and public health research interested in disparities by race or ethnicity.

## Methods

The research was predominantly led by the first four co-authors who identify as Pākehā or European men, some of whom self-identify as gay. The remaining co-authors identify with a variety of ethnicities. New Zealand’s HIV behavioural surveillance among MSM consists of repeated cross-sectional surveys conducted in multiple settings using consistent sampling over time: the Gay Auckland Periodic Sex Survey (GAPSS) and Gay men’s Online Sex Survey (GOSS). In 2006, 2008, 2011 and 2014, GAPSS participants were recruited to self-complete voluntary and anonymous questionnaires from a community fair day, gay bars, and sex-on-site venues in Auckland (New Zealand’s largest city), and GOSS participants from online dating sites nationally. Eligible men had to be at least 16 years old and report sex with a man in the last 5 years, and had not completed the survey previously that round. Responses from all four rounds were pooled to increase statistical power, and individuals who indicated participation in a previous year were removed in order to satisfy statistical assumptions of independent observations; this resulted in each participant being represented once in the dataset based on their earliest response. The Northern X Regional Ethics Committee granted ethical approval. Detailed surveillance methods are reported elsewhere [2].

Ethnicity, the primary independent variable, was self-identified by participants using the Statistics New Zealand’s 2013 census question (Table 1), asked in both English and te reo Māori. Results for the four largest ethnic groups are reported in alphabetical order: Asian (including Chinese and Indian), European, Indigenous Māori (the indigenous people of Aotearoa New Zealand), and Pacific (including Samoan, Cook Island Māori, Tongan, and Niuean) people. Importantly, people could indicate more than one ethnicity, without a requirement to indicate a sole primary or main one. Three alternative ethnicity classification systems to deal with the multiple responses data from “multi-ethnic” participants were applied: prioritisation, single/combination, and total response.

**Table 1:** Question on Ethnic group in the 2013 New Zealand Census of Population and Dwellings, Individual Form (only shown in English language)

Which ethnic group do you belong to? *Mark the space or spaces which apply to you.*	
o New Zealand European o Māori o Samoan o Cook Island Māori o Tongan o Niuean o Chinese o Indian o Other such as *DUTCH, JAPANESE, TOKELAUAN.* Please state: ______________________________ ______________________________ ______________________________	

### Prioritisation

If an individual reported multiple ethnicities they were categorised into a single discrete group and prioritised in the following hierarchy: first, Māori; second, Pacific; third, Asian; fourth, Other, and last, European (typically used as the referent group). For example, if someone identified as Māori and any other ethnicity, they were classified as Māori; if someone identified as Pacific and any other ethnicity except Māori, they were classified as Pacific; and if someone identified as Asian and any other ethnicity except Māori or Pacific, they were classified as Asian. Only those who identified as New Zealand European and/or any other European ethnicity were identified as European. This classification system produces a single ethnicity variable with no ability to identify multi-ethnic participants. Participants were coded as 1 “Māori”, 2 “Pacific”, 3 “Asian”, 4 “Other” or 0 “European”.

### Single/combination

Similar to prioritisation, an individual is assigned to a discrete group within a single variable, but this method explicitly specifies multiple ethnicity combinations (e.g., European-Māori, Asian-Pacific) alongside exclusive major ethnicities (e.g., only Māori, only Pacific). This approach allows for identification of multi-ethnic participants as one or several discrete groups/levels in a single variable. This method adheres better to principles of self-identity for participants who report multiple ethnicities, but can be hampered by the large number of possible ethnic combinations and limited counts for multiple combination groups. There is also a highly heterogeneous category of multiple ethnicity remainder participants from ethnic combinations not explicitly reported as separate levels of this variable. Participants were coded as 1 “Māori only”, 2 “Pacific only”, 3 “Asian only”, 4 “Other only”, 5 “Māori-European”, 6 “Pacific-European, 7 “Other ≥ 2 combinations”, or 0 “European only”.

### Total response

Separate variables are established for each major ethnic group and an individual may be included in more than one of these. For example, a respondent identifying with both Māori and Pacific ethnicities would be included in two variables, both the Māori and the Pacific variables. Referent categories then become all other participants who did not indicate that ethnicity (e.g., Māori versus non-Māori). This multiple variable approach does not provide an explicit identification of multi-ethnic participants. In our analysis we use a common “European-only” comparator group for each major ethnicity variable (i.e., Māori versus European-only, Pacific versus European-only, and Asian versus European-only), similar to previously published ethno-specific analyses of hospital intake data [11]. This “modified total response” approach enables comparisons with the ethnic group of greatest population size and social power (i.e. European). It also reduces heterogeneity of the referent group by removing Indigenous Māori and minority ethnicities that might otherwise obfuscate between-group differences. The use of a modified total response approach also means that all three classification methods have a consistent “European-only” referent. Participants were coded into a different variable for each major ethnic group: Māori variable coded as 1 “Māori”, 0 “European-only”, and 99 for “neither Māori nor European-only”, Pacific variable coded as 1 “Pacific”, 0 “European-only”, and 99 for “neither Pacific nor European-only”, etc.

Full descriptions of each method can be found elsewhere [9]. However, prioritisation is noteworthy because although this method has been discontinued in official government statistics as of 2005 as it under-counted Pacific and other minority ethnicities and had purported inadequate theoretical basis, [12] it is still commonly used in the health sector particularly when drawing comparisons between groups [13]. HIV behavioural surveillance in New Zealand has also continued to use this method [2]. Indigenous Māori perspectives on ethnicity classification include their right to name individual and collective identities [14]. We acknowledge and support the rights to self-identify and self-determination, which should be central to any work examining ethnicity.

The dependent variables used to compare methods included recruitment method (online versus in-person), three demographic factors (age < 30 years versus older, gay identity versus other, and at least some tertiary education versus none), and eight sexual health indicators (greater than 20 male sexual partners in the 6 months prior to survey, STI testing or treatment in the year prior to survey, any STI diagnosis in the year prior to survey, lifetime HIV testing, HIV testing in the year prior to survey, any HIV positive test result, any condomless anal intercourse (CAI) with casual partners in the 6 months prior to survey, and any condomless anal intercourse with regular partners in the six months prior to survey). Casual partners were defined as someone the participant had sex with up to three times in the 6 months prior to survey; regular partners were those they had sex with four or more times in the last 6 months.

All statistical analyses were conducted using Stata/SE 13.1. We compared the recruitment method and demographics of respondents who completed the ethnicity question with those who did not using chi-square tests of independence. Descriptive statistics for each variable were prepared based on each of the three ethnicity classification methods. Each demographic and sexual health outcome was regressed on ethnicity separately for each classification method described above using univariate logistic regression (*p* < 0.05 considered significant). Multivariable logistic regression was used to analyse further differences in sexual health outcomes after controlling for recruitment and demographic factors (i.e., recruitment year and method, age, education, and sexual identity), which were all forced into the models. Adjusted odds ratios (AOR) and 95% confidence intervals (95% CI) are shown.

## Results

A total of 12,816 questionnaires were completed across the 2006, 2008, 2011 and 2014 national HIV behavioural surveillance rounds. Of these, 1896 (14.8%) questionnaires were removed as they indicated completion of an earlier survey. Of 10,920 unique responses, 10,525 MSM completed the ethnicity question (*n* = 395, 3.6% missing). Men who did not complete the ethnicity question were more likely to have participated in-person than online (*p* < 0.001) and to have some tertiary/post-secondary education than none (*p* = 0.003); there was no difference in missing ethnicity data by age (*p* = 0.261) or sexual identity (*p* = 0.480).

The sample size for each major ethnic group varied by classification method (Table 2). Multiple ethnicities were reported by 762 (7.2%) participants. All three classification systems identify the same number of individuals as European-only (*n* = 7867, 74.7% of respondents), which was the referent group used for comparisons within each classification method. Using Māori ethnicity as an example, all respondents who reported being Māori (*n* = 1058, 10.1%) were categorised as Māori for *Prioritisation* and *Total Response* classification, and since Māori have first priority for classification in *Prioritisation* the sample size of Māori is identical for both classification methods (10.1%)*.* However, for *Single/Combination*, individuals who reported being Māori and no other ethnicity (*n* = 538, 5.1% of the sample or 50.9% of all Māori-identifying respondents) were categorised into a Māori only group, with the remaining 520 respondents (4.9%) reporting Māori and another ethnicity (most of whom reported being Māori and European, *n* = 428, 4.1%). The next largest and only other pairwise ethnic combination reported for *Single/Combination* was European-Pacific (*n* = 111, 1.1%). The remaining respondents who indicated any other combination of major ethnic groups (*n* = 223) were grouped together because there were fewer than 100 individuals (< 1% of entire sample) in each category.

**Table 2:** Sample size of major ethnic groups in New Zealand by three classification methods (*n* = 10,525)

	Prioritisation		Single/Combination		Total Response	
	n	%	n	%	n	%
Asian	944	9.0	875^a^	8.3	1003	9.8
European (only)^a^	7867^a^	74.7	7867^a^	74.7	7867^a^	74.7
Māori	1058	10.1	538^a^	5.1	1058	10.1
Other	305	2.9	282^a^	2.7	325	3.1
Pacific	351	3.3	201^a^	1.9	424	4.0
European-Māori	N/A		428	4.1	N/A	
European-Pacific	N/A		111	1.1	N/A	
Other ≥2 combinations	N/A		223	2.1	N/A	

*NB* Percentages sum to more than 100% for Total Response as groups are not mutually exclusive

*N/A* not applicable to that classification method

^a^Denotes sole major ethnic group

Differences by recruitment method, age, sexual identity and educational attainment between ethnic groups and across the classification methods are shown in Table 3. Regardless of classification method, compared with European MSM, Māori MSM and ethnic minority MSM were less likely to be recruited online and more likely to be younger with the exception of Māori-only MSM using the Single/Combination classification, which did not have a statistically significant difference by age. Gay identity was more likely among Asian MSM, less likely among Pacific and Māori MSM, and no difference was seen for MSM of other ethnicities. When compared with European MSM, Māori and Pacific MSM were less likely to report a tertiary degree while Asian and Other MSM were more likely to report one.

**Table 3:** Recruitment and demographic differences between major ethnic groups in New Zealand within each of three different classification methods (*n* = 10,525)

	Ethnicity	Prioritisation *n* (%)	Single/Combination *n* (%)	Total Response *n* (%)
Recruitment (online)	European (referent)	5068 (64.4)	5068 (64.4)	5068 (64.4)
	Māori	646 (61.1)*	301 (56.0)*	646 (61.1)*
	Pacific	185 (52.7)*	95 (47.3)*	222 (52.4)*
	Asian	453 (48.0)*	421 (48.1)*	490 (48.9)*
	Other	131 (43.0)*	121 (42.9)*	141 (43.4)*
	European-Māori		299 (69.9)*	
	European-Pacific		65 (58.6)	
	Other combinations		113 (50.7)*	
Age (less than 30 years)	European (referent)	3086 (39.5)	3086 (39.5)	3086 (39.5)
	Māori	549 (52.8)*	232 (43.8)	549 (52.8)*
	Pacific	218 (62.8)*	118 (59.6)*	264 (63.2)*
	Asian	543 (58.0)*	497 (57.2)*	582 (58.6)*
	Other	136 (45.2)*	127 (45.7)*	149 (46.6)*
	European-Māori		262 (62.4)*	
	European-Pacific		73 (65.8)*	
	Other combinations		137 (62.8)*	
Identity (gay)	European (referent)	5690 (72.6)	5690 (72.6)	5690 (72.6)
	Māori	657 (62.4)*	321 (60.0)*	657 (62.4)*
	Pacific	214 (61.3)*	107 (53.8)*	258 (61.1)*
	Asian	739 (78.8)*	681 (78.4)*	775 (77.7)*
	Other	228 (75.5)	208 (74.3)	242 (75.2)
	European-Māori		279 (65.5)*	
	European-Pacific		83 (74.8)	
	Other combinations		159 (71.6)	
Education (tertiary degree or higher)	European (referent)	2862 (36.8)	2862 (36.8)	2862 (36.8)
	Māori	335 (32.1)*	159 (30.0)*	335 (32.1)*
	Pacific	109 (31.8)*	59 (30.0)*	135 (32.5)*
	Asian	637 (68.1)*	605 (69.8)*	667 (67.2)*
	Other	177 (58.8)*	162 (58.3)*	182 (57.4)*
	European-Māori		143 (33.7)	
	European-Pacific		30 (27.5)	
	Other combinations		100 (45.7)	

* Statistically significant difference with European category based on univariate logistic regression (*p* < 0.05)

Descriptive statistics and univariate differences by ethnicity are shown in Table 4. Overall, 932 men (9.1%) reported having more than 20 male sexual partners in the past 6 months, 2828 men (27.8%) reported CAI with a *casual* partner in the past 6 months, and 2617 men (25.7%) reported CAI with a *regular* partner in the past 6 months. Further, 4858 men (48.9%) reported STI testing in the past year, and 977 men (9.5%) reported an STI diagnosis in the past year. With respect to HIV, 7022 men (67.3%) reported an HIV test in their lifetime, 4185 men (40.1%) reported an HIV test in the past year, and 316 men (3.1%) reported an HIV diagnosis. There were univariable differences by ethnicity for each outcome.

**Table 4:** Sexual health outcomes of major ethnic groups in New Zealand by three classification methods

	Ethnicity	Prioritisation *n* (%)	Single/Combination *n* (%)	Total response *n* (%)
Number of sexual partners (greater than 20 in past 6 months)	European (referent)	666 (8.7)	666 (8.7)	666 (8.7)
	Māori	112 (10.9)*	59 (11.4)*	112 (10.9)*
	Pacific	47 (13.8)*	27 (14.0)*	55 (13.4)*
	Asian	76 (8.2)	69 (8.0)	84 (8.5)
	Other	31 (10.3)	28 (10.0)	33 (10.3)
	European-Māori		43 (10.3)	
	European-Pacific		14 (12.8)	
	Other combinations		26 (12.1)	
Condomless anal intercourse with *casual* partner (any in past 6 months)	European (referent)	2115 (27.7)	2115 (27.7)	2115 (27.7)
	Māori	322 (31.9)*	154 (30.4)	322 (31.9)*
	Pacific	108 (32.0)	61 (31.4)	128 (31.3)
	Asian	214 (23.5)*	199 (23.6)*	233 (24.0)*
	Other	69 (23.2)	66 (23.9)	74 (23.4)
	European-Māori		141 (34.1)*	
	European-Pacific		37 (34.9)	
	Other combinations		55 (25.6)	
Condomless anal intercourse with *regular* partner (any in past 6 months)	European (referent)	2008 (26.3)	2008 (26.3)	2008 (26.3)
	Māori	224 (22.2)*	115 (22.5)	224 (22.2)*
	Pacific	95 (28.3)	58 (30.7)	114 (28.1)
	Asian	209 (22.9)*	194 (22.9)*	223 (23.0)*
	Other	81 (26.9)	73 (26.2)	85 (26.6)
	European-Māori		86 (20.9)*	
	European-Pacific		29 (26.6)	
	Other combinations		54 (25.6)	
STI testing or treatment (past 12 months)	European (referent)	3671 (47.3)	3671 (47.3)	3671 (47.3)
	Māori	513 (49.6)	260 (49.9)	513 (49.6)
	Pacific	148 (43.8)	83 (43.7)	185 (45.1)
	Asian	376 (40.3)*	341 (39.4)*	402 (40.6)*
	Other	150 (49.7)	138 (49.5)	161 (50.0)
	European-Māori		207 (48.8)	
	European-Pacific		47 (43.1)	
	Other combinations		111 (50.5)	
STI diagnosis (past 12 months)	European (referent)	740 (9.7)	740 (9.7)	740 (9.7)
	Māori	104 (10.2)	50 (9.8)	104 (10.2)
	Pacific	43 (12.7)	23 (12.0)	58 (14.1)*
	Asian	68 (7.5)*	60 (7.2)*	79 (8.2)
	Other	22 (7.3)	22 (7.9)	25 (7.8)
	European-Māori		35 (8.3)	
	European-Pacific		15 (13.6)	
	Other combinations		32 (14.6)*	
HIV testing (ever)	European (referent)	5306 (68.0)	5306 (68.0)	5306 (68.0)
	Māori	683 (65.2)	348 (65.7)	683 (65.2)
	Pacific	185 (53.9)*	98 (50.5)*	231 (55.7)*
	Asian	604 (64.8)*	557 (64.5)*	632 (63.8)*
	Other	244 (80.8)*	224 (80.3)*	255 (79.2)*
	European-Māori		276 (64.6)	
	European-Pacific		72 (65.5)	
	Other combinations		141 (63.5)	
HIV testing (past 12 months)	European (referent)	3111 (39.9)	3111 (39.9)	3111 (39.9)
	Māori	433 (41.3)	212 (40.0)	433 (41.3)
	Pacific	114 (33.2)*	59 (30.4)*	144 (34.7)*
	Asian	374 (40.1)	345 (40.0)	392 (39.6)
	Other	153 (50.7)*	141 (50.5)*	161 (50.0)*
	European-Māori		183 (42.9)	
	European-Pacific		45 (40.9)	
	Other combinations		89 (40.1)	
HIV positive test result (ever)	European (referent)	262 (3.4)	262 (3.4)	262 (3.4)
	Māori	19 (1.9)*	10 (1.9)	19 (1.9)*
	Pacific	10 (3.0)	5 (2.7)	13 (3.2)
	Asian	12 (1.3)*	11 (1.3)*	14 (1.4)*
	Other	13 (4.4)	13 (4.7)	14 (4.4)
	European-Māori		4 (1.0)*	
	European-Pacific		4 (3.7)	
	Other combinations		7 (3.2)	

*statistically significant difference based on univariate logistic regression (*p*<0.05)

Table 5 presents the multivariable differences between sexual health outcomes by ethnicity classification method, which controlled for recruitment method and year, age, sexual identity, and education. The patterns of differences between ethnic groups, or lack thereof, were similar across all classification methods for six of the sexual health outcomes: number of sexual partners, any condomless anal intercourse with casual partners, recent STI testing, lifetime and recent HIV testing, and HIV status. However, associations between ethnicity and two sexual health outcomes (i.e. any recent condomless anal intercourse with regular partners and recent STI diagnoses) varied by ethnicity classification system.

**Table 5:** Sexual health outcomes of major ethnic groups in New Zealand by three classification methods controlling for recruitment year, method, and demographics

	Ethnicity	Prioritisation *AOR* (95% CI)	Single/Combination *AOR* (95% CI)	Total response *AOR* (95% CI)
Number of sexual partners (greater than 20 in past 6 months)	European (referent)	1.00	1.00	1.00
	Māori	**1.46 (1.17-1.81)**	**1.41 (1.05-1.89)**	**1.46 (1.17-1.81)**
	Pacific	**2.12 (1.53-2.93)**	**2.22 (1.45-3.39)**	**2.05 (1.52-2.78)**
	Asian	1.04 (0.80-1.34)	1.01 (0.78-1.32)	1.09 (0.86-1.40)
	Other	1.26 (0.85-1.86)	1.23 (0.82-1.86)	1.29 (0.88-1.89)
	European-Māori		**1.47 (1.05-2.05)**	
	European-Pacific		**1.86 (1.05-3.30)**	
	Other combinations		**1.73 (1.13-2.64)**	
Condomless anal intercourse with *casual* partner (any in past 6 months)	European (referent)	1.00	1.00	1.00
	Māori	**1.28 (1.11-1.49)**	**1.26 (1.02-1.54)**	**1.28 (1.11-1.49)**
	Pacific	**1.44 (1.13-1.84)**	**1.50 (1.09-2.06)**	**1.42 (1.13-1.77)**
	Asian	0.92 (0.78-1.09)	0.93 (0.78-1.11)	0.96 (0.81-1.13)
	Other	0.95 (0.71-1.26)	0.99 (0.74-1.33)	0.97 (0.74-1.28)
	European-Māori		**1.28 (1.03-1.60)**	
	European-Pacific		1.46 (0.96-2.21)	
	Other combinations		1.07 (0.77-1.47)	
Condomless anal intercourse with *regular* partner (any in past 6 months)	European (referent)	1.00	1.00	1.00
	Māori	**0.84 (0.71-0.99)**	0.84 (0.68-1.05)	**0.84 (0.71-0.99)**
	Pacific	1.20 (0.93-1.54)	1.38 (0.99-1.91)	1.18 (0.94-1.49)
	Asian	**0.79 (0.67-0.94)**	**0.79 (0.66-0.94)**	**0.80 (0.68-0.94)**
	Other	0.99 (0.76-1.30)	0.96 (0.73-1.27)	0.99 (0.76-1.29)
	European-Māori		0.78 (0.61-1.01)	
	European-Pacific		1.03 (0.68-1.59)	
	Other combinations		1.01 (0.73-1.39)	
STI testing or treatment (past 12 months)	European (referent)	1.00	1.00	1.00
	Māori	1.10 (0.96-1.26)	1.14 (0.95-1.37)	1.10 (0.96-1.26)
	Pacific	0.88 (0.70-1.10)	0.89 (0.66-1.20)	0.92 (0.75-1.14)
	Asian	**0.67 (0.58-0.78)**	**0.65 (0.56-0.75)**	**0.68 (0.59-0.79)**
	Other	1.00 (0.79-1.27)	0.98 (0.77-1.26)	1.03 (0.82-1.30)
	European-Māori		1.04 (0.85-1.28)	
	European-Pacific		0.84 (0.57-1.23)	
	Other combinations		1.08 (0.82-1.43)	
STI diagnosis (past 12 months)	European (referent)	1.00	1.00	1.00
	Māori	1.09 (0.87-1.36)	1.07 (0.79-1.46)	1.08 (0.87-1.35)
	Pacific	1.40 (1.00-1.96)	1.29 (0.82-2.04)	**1.61 (1.20-2.16)**
	Asian	**0.69 (0.53-0.90)**	**0.66 (0.49-0.87)**	**0.77 (0.59-0.99)**
	Other	0.69 (0.44-1.09)	0.75 (0.47-1.18)	0.76 (0.49-1.16)
	European-Māori		0.84 (0.59-1.21)	
	European-Pacific		1.52 (0.87-2.65)	
	Other combinations		**1.61 (1.09-2.38)**	
HIV testing (ever)	European (referent)	1.00	1.00	1.00
	Māori	1.07 (0.92-1.24)	1.03 (0.84-1.26)	1.07 (0.92-1.24)
	Pacific	**0.69 (0.54-0.88)**	**0.60 (0.43-0.82)**	**0.75 (0.60-0.93)**
	Asian	**0.72 (0.62-0.85)**	**0.70 (0.60-0.83)**	**0.71 (0.61-0.83)**
	Other	**1.69 (1.24-2.32)**	**1.64 (1.19-2.26)**	**1.61 (1.19-2.17)**
	European-Māori		1.12 (0.90-1.39)	
	European-Pacific		1.14 (0.74-1.75)	
	Other combinations		0.92 (0.68-1.26)	
HIV testing (past 12 months)	European (referent)	1.00	1.00	1.00
	Māori	1.08 (0.95-1.24)	1.06 (0.88-1.28)	1.09 (0.95-1.24)
	Pacific	0.80 (0.63-1.02)	0.73 (0.53-1.01)	0.85 (0.68-1.05)
	Asian	**0.85 (0.73-0.98)**	**0.84 (0.72-0.97)**	**0.84 (0.73-0.96)**
	Other	**1.31 (1.03-1.67)**	**1.30 (1.01-1.66)**	**1.32 (1.04-1.66)**
	European-Māori		1.11 (0.91-1.37)	
	European-Pacific		1.04 (0.70-1.53)	
	Other combinations		0.98 (0.74-1.30)	
HIV positive test result (ever)	European (referent)	1.00	1.00	1.00
	Māori	0.70 (0.44-1.13)	0.63 (0.33-1.21)	0.70 (0.44-1.13)
	Pacific	1.34 (0.69-2.58)	1.25 (0.50-3.11)	1.50 (0.84-2.67)
	Asian	**0.39 (0.21-0.72)**	**0.38 (0.20-0.73)**	**0.44 (0.25-0.79)**
	Other	1.06 (0.57-2.00)	1.16 (0.61-2.19)	1.12 (0.61-2.06)
	European-Māori		0.41 (0.15-1.12)	
	European-Pacific		1.47 (0.52-4.11)	
	Other combinations		1.35 (0.62-2.95)	

**Bolded text** indicates statistical significance based on multivariable logistic regression (*p*<0.05)

First, Māori MSM were significantly less likely to report condomless anal intercourse with a regular partner in the past 6 months compared with European MSM when using *Prioritisation* (AOR = 0.84, 95%CI: 0.71–0.99) or *Total Response* (AOR = 0.84, 95%CI: 0.71–0.99), but not significantly different statistically when using *Single/Combination* (AOR = 0.84, 95%CI: 0.68–1.05). Of note, these AOR are the same across ethnicity classification methods, but have different levels of precision. Second, compared with European MSM, Pacific MSM were significantly more likely to report a recent STI diagnosis when using *Total Response* (AOR = 1.61, 95%CI: 1.20–2.16), with no statistically significant differences when using *Prioritisation* (AOR = 1.40, 95%CI: 1.00–1.96) or *Single/Combination* (AOR = 1.29, 95%CI: 0.82–2.04). Of note, these AOR are similar across ethnicity classification methods, with different levels of precision.

There were some differences for participants who reported multiple ethnicities when compared with European-only participants. For example, when identified using *Single/Combination,* multi-ethnic participants were significantly more likely to report greater than 20 sexual partners in the past 6 months (AOR = 1.73, 95%CI: 1.13–2.64) and to report an STI diagnosis in the past year (AOR = 1.61 95%CI: 1.09–2.38).

## Discussion

Using a pooled sample of 10,525 respondents who reported an ethnicity within the 2006–2014 rounds of New Zealand’s national HIV behavioural surveillance for MSM, we compared three ethnicity classification methods (Prioritisation, Single/Combination and Total Response) and their impact on sample size, demographics, and sexual health outcomes. Different classification methods to categorize participants who reported multiple ethnicities altered sample size, and also revealed and masked associations in two of the eight selected sexual health outcomes by ethnicity. For example, even after controlling for demographic differences, when compared with European MSM, Prioritisation and Total Response ethnicity classification methods showed statistically significant higher condom use with regular partners among Māori MSM, while Single/Combination classification limited the sample size resulting in no observed statistically significant difference. However, for recent STI diagnoses among Pacific MSM, Total Response classification system, which maximised sample size, demonstrated a statistically significant difference and higher odds ratio when compared with European men while Prioritisation and Single/Combination classifications resulted in no statistically significant differences. The Single/Combination classification approach highlighted specific differences for multi-ethnic participants compared with European-only participants such as greater odds of higher recent sexual partner numbers and recent STI diagnoses. For the remaining six sexual health outcomes examined, different classification methods resulted in consistent patterns of association.

Given the discontinued use of *prioritisation* as an official government method of ethnicity classification in New Zealand, [9] a decision that was opposed by Indigenous Māori based on their rights to name individual and collective identities, [14] our study aimed to determine the impact of different classification methods when participants select multiple ethnic identities. Each classification method has its own strengths and drawbacks, which range from socio-political to technical statistical issues. Prioritisation and Single/Combination produce single ethnicity variables that are simpler to report on and include in regression analyses. Ultimately, the choice of classification system should stem from the research question and population focus. If focused on overall populations and equity, we assert that prioritisation provides a single variable, upholds Indigenous Māori rights to self-identify, but undercounts Pacific and Asian participants. Total Response follows tenets of self-determination for all groups by assigning participants to each major ethnic group with which they identify, thus producing individual variables for each major ethnic group. For within-group/specific ethnic group analyses (e.g. among Pacific MSM exclusively), Total Response allows all participants who identified with a specific ethnicity to be counted as such. This will ensure that Pacific and Asian participants will have the most complete and accurate data possible to inform targeted strategies and programmes.

Classification method decisions will impact sample size; classification methods that produce the largest possible sample size for Indigenous Māori (Prioritisation and Total Response) and ethnic minority groups (Total Response) minimize the chance of Type II “missed opportunity” errors as long as differential or non-differential misclassification is not increased, which is crucial given public health’s interest in the experiences of and equity for Indigenous and minority groups. Future research, especially that interested in ethnic/racial inequities, should strive for equal explanatory power to ensure adequate sample sizes of Indigenous and ethnic minority groups to answer research questions [15]. Developed for Indigenous Māori, these approaches provide appropriate sampling methodology, respect for community voices, and are feasible in general and sub-population studies [16, 17].

A major strength of the current analyses was the comparison of three ethnicity classification approaches in a country with a strong history of public health interest in ethnicity. We pooled data across four cycles of an established national HIV surveillance programme providing large samples [2]. We examined behavioural data, extending previous research beyond hospital records [11]. We used multivariable analyses of sexual health outcomes to control for distinct recruitment and demographic differences by ethnicity. In the current study, Māori and Pacific men (as well as European-Māori and European-Pacific men) were more likely to report greater than 20 male sexual partners compared with European men, which may increase the likelihood of reporting *any* condomless anal intercourse given the increased number of sexual partners, highlighting the sensitivity of using such measures of risk. Data were self-reported, which limits our ability to examine actual HIV status. However, the indicators considered in our study address a causal pathway from risk behaviour (number of sexual partners and condom use) to health service use (HIV/STI testing) to health outcome (STI and HIV diagnoses). It is critically important that future work examine the temporal trends in these behavioural and health service outcomes, along with other structural factors which causally precede HIV exposure and transmission.

Previous analyses of MSM in New Zealand have considered ethnicity, but not as the primary explanatory variable or outcome. An exception is the 1996 Male Call/Waea Mai, Tāne Mā report, which compared Māori with non-Māori participants [18]. Compared with findings for Māori men from this 1996 national telephone survey of MSM, [18] the current study identified a persistent difference that Māori men were less likely to report condomless anal intercourse with regular partners. The current study identified two previously undocumented differences for Māori men, which were a greater likelihood of reporting greater than 20 sexual partners and any condomless anal intercourse with casual partners. Compared with European men in the current study, Pacific men were more likely to report greater than 20 partners and more likely to report any condomless anal intercourse with casual partners, the latter corroborating an analysis of infrequent condom use using 2014 data [19]. Other cross-sectional research focused on ethnic minority MSM in Britain [20] and the United Kingdom [21] found no effect by ethnicity with regard to reports of condomless anal intercourse and cross-sectional research in China found that some ethnic minority MSM were less likely to report condomless anal sex [22].

With regards to HIV testing, Pacific MSM in the current study were less likely to have ever had an HIV test, an HIV testing disparity not present in the 1996 Male Call study in New Zealand [23], but noted in a research brief using the 2014 behavioural surveillance data [24]. Asian MSM in the current study were less likely to report any lifetime HIV testing and being HIV-positive, and also less likely to report any recent STI testing or diagnosis. The proportion of Asian MSM who reported never having been tested for HIV (35%) was higher than the proportion reported in the metro United States (8%) [25], Britain (Chinese: 23%, South Asian: 32%) [21], or Canada (21%) [26]. Previous studies had not found differences for Asian MSM in the United Kingdom [21] or in Canada [26] with respect to HIV testing uptake.

Public health and biomedical research has generally been poor at providing adequate detail about how race and/or ethnicity is operationalised [27]. Greater transparency is essential to improve confidence that public health responses are based on fair and rigorous data processes that can identify inequities [28]. Approaches to ethnicity classification should also be primarily driven by the research or public health objectives, particular if they relate to Indigenous and ethnic minority identities and communities [13]. Measures of ethnicity should include locally relevant categories, which may be aggregated into broader categories given practical constraints of population-based questionnaires (e.g., limited counts in some sub-groups) [28]. However, the meaning and utility of any macro-ethnic groupings must be questioned for programming and policy (e.g., Samoan and Tongan persons combined together into Pacific people [29], diverse groups combined as Asian people [30]). Further, in the context where the current study took place an individual’s ethnicity is not an ingrained or permanent trait, but is instead influenced by social and environmental factors over time [31].

Ethnicity-focused research must also consider connections with other identities. For example, how sexuality may be influenced by and mutually reinforce constructs of ethnicity, and vice versa [32]. Intersectionality scholars push further and challenge public health and researchers to consider also the meaning of categories, more complex social locations (e.g., interactions with social class, age, gender, immigration status, acculturation), and social and behavioural processes [33, 34]. Research should acknowledge both the characteristics and attributes of varying ethnicities, but also the social processes and hierarchies between ethnic groups [31]. This approach may be more relevant to informing health promotion and interventions aimed at achieving health equity by ethnic affiliation [33, 34]. For example, previous work has demonstrated the added value of examining socially-assigned ethnicity to expand understandings of racialization and racism as a social determinant of health [35, 36]. This may reduce issues of misclassification bias introduced when ethnic identity data are used as a proxy for these social processes and experiences. Future research would benefit from the inclusion of measures beyond ethnic/racial identity to those such as socially-assigned race/ethnicity and experiences of discrimination and racism.

## Conclusions

In conclusion, our analyses evaluated ethnicity classification methods and commented on the implications for public health equity and research. As the identification of inequities can activate public health responses, but also (re) produce stigma, our findings have implications for government officials, policy makers, funders, and community stakeholders. While these analyses draw on pooled data specifically related to sexual health, HIV/STIs, and the MSM population, the implications of this work are pertinent to any medical or public health work examining inequities by ethnicity.

## Data Availability

The 2006–2014 GAPSS and GOSS datasets were used and analysed during the current study and are available on reasonable request and with permission from Dr. Peter JW Saxton (p.saxton@auckland.ac.nz).

## Abbreviations

HIV: Human immunodeficiency virus
MSM: Men who have sex with men
STI: Sexually transmitted infections
GAPSS: Gay Auckland Periodic Sex Survey
GOSS: Gay men’s Online Sex Survey
CAI: Condomless anal intercourse
AOR: Adjusted odds ratios
CI: Confidence intervals
